# Supplementary material for: The interactome of intact mitochondria by cross-linking mass spectrometry provides evidence for coexisting respiratory supercomplexes
Source: Mol Cell Proteomics. 2017 Dec 8;17(2):216–32. doi: 10.1074/mcp.RA117.000470 (PMC5795388; doi:10.1074/mcp.RA117.000470)
Supplement: Supplemental Data [file supp_RA117.000470_133922_0_supp_23978_fzffwf.zip › spectra_annotation/mito_DR_spectra_annotation/208-1-8-1-4-1.pdf]

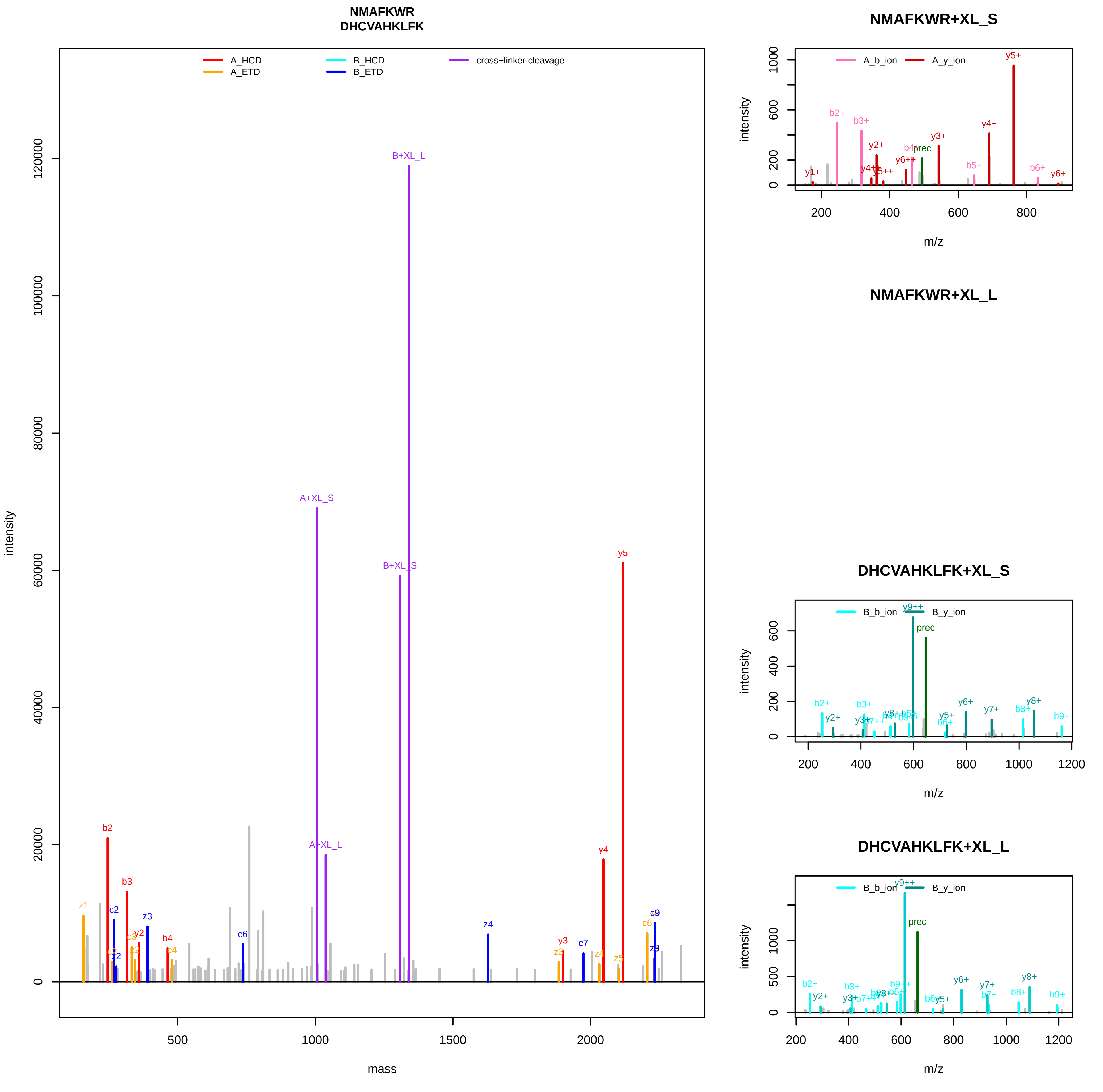
NMAFKWR
DHCVAHKLFK
A_HCD
A_ETD
B_HCD
B_ETD
cross−linker cleavage
intensity
mass
0
20000
40000
60000
80000
100000
120000
500
1000
1500
2000
B+XL_L
A+XL_S
B+XL_S
A+XL_L
b2
b3
b4
y2
y3
y4
y5
z1
c2
z2
c3
z3
c4
c6
c7
z4
z5
c9
z9
NMAFKWR+XL_S
A_b_ion
A_y_ion
m/z
b2+
b3+
b4+
b5+
b6+
y1+
y2+
y3+
y4+
y5+
y6+
y4++
y5++
y6++
prec
NMAFKWR+XL_L
DHCVAHKLFK+XL_S
B_b_ion
B_y_ion
y9++
b2+
b3+
b8+
b9+
y2+
y3+
y5+
y6+
y7+
y8+
DHCVAHKLFK+XL_L
0
200
400
600
800
1000
1200
